# MG-MLST: Characterizing the Microbiome at the Strain Level in Metagenomic Data

**DOI:** 10.3390/microorganisms8050684

**Published:** 2020-05-08

**Authors:** Nathanael J. Bangayan, Baochen Shi, Jerry Trinh, Emma Barnard, Gabriela Kasimatis, Emily Curd, Huiying Li

**Affiliations:** 1 Department of Molecular and Medical Pharmacology, Crump Institute for Molecular Imaging, University of California, Los Angeles, CA 90095, USA; NBangayan@mednet.ucla.edu (N.J.B.); biosbc@gmail.com (B.S.); jtrinh8@ucla.edu (J.T.); emmab11@hotmail.co.uk (E.B.); gabi.kasimatis@gmail.com (G.K.); eecurd@ucla.edu (E.C.); 2 Department of Ecology and Evolutionary Biology, University of California, Los Angeles, CA 90095, USA; 3 UCLA-DOE Institute for Genomics and Proteomics, Los Angeles, CA 90095, USA

**Keywords:** microbiome, strain, metagenomics, MLST, *Propionibacterium acnes*, method

## Abstract

The microbiome plays an important role in human physiology. The composition of the human microbiome has been described at the phylum, class, genus, and species levels, however, it is largely unknown at the strain level. The importance of strain-level differences in microbial communities has been increasingly recognized in understanding disease associations. Current methods for identifying strain populations often require deep metagenomic sequencing and a comprehensive set of reference genomes. In this study, we developed a method, metagenomic multi-locus sequence typing (MG-MLST), to determine strain-level composition in a microbial community by combining high-throughput sequencing with multi-locus sequence typing (MLST). We used a commensal bacterium, *Propionibacterium acnes*, as an example to test the ability of MG-MLST in identifying the strain composition. Using simulated communities, MG-MLST accurately predicted the strain populations in all samples. We further validated the method using MLST gene amplicon libraries and metagenomic shotgun sequencing data of clinical skin samples. MG-MLST yielded consistent results of the strain composition to those obtained from nearly full-length 16S rRNA clone libraries and metagenomic shotgun sequencing analysis. When comparing strain-level differences between acne and healthy skin microbiomes, we demonstrated that strains of RT2/6 were highly associated with healthy skin, consistent with previous findings. In summary, MG-MLST provides a quantitative analysis of the strain populations in the microbiome with diversity and richness. It can be applied to microbiome studies to reveal strain-level differences between groups, which are critical in many microorganism-related diseases.

## 1. Introduction

Our knowledge of the human microbiome and its relationship to health and disease has been rapidly increasing in recent years. Many studies have characterized the microbial communities at various sites of the human body, including the gut, the oral cavity, the urogenital tract, the respiratory tract, and the skin. However, most of the microbiome studies have only been able to characterize the bacterial communities to the genus or species level, leaving the microbiome composition at the strain level largely undefined. On the other hand, many studies have shown that strain-level differences of a microorganism are important in determining its beneficial or pathogenic potential to the host. For example, within the species of *Escherichia coli*, strain Nissle 1917 has been used as a probiotic to treat ulcerative colitis [1], while strain O157:H7 is the most common cause of hemolytic uremic syndrome [2]. Another example is *Propionibacterium acnes*, a common commensal found on the human skin. Certain strains of *P. acnes* have been associated with the disease acne vulgaris, while other strains have been associated with healthy skin [3,4,5]. By studying the strain composition of the microbiome, new correlations or causal relationships between microbial organisms and health or disease may be discovered.

Currently, high-throughput 16S ribosomal RNA (rRNA) sequencing is the most common method to study the bacterial composition of a community. However, in most cases it is limited in its ability to detect microorganisms at the strain level due to the resolution provided by the hypervariable regions of the 16S rRNA gene. Although metagenomic shotgun sequencing can provide strain-level information [6], it presents challenges in that it often requires deep sequencing and a comprehensive set of reference genomes to distinguish selected strains. A potential alternative approach to study the microbiome at the strain level is to employ multi-locus sequence typing (MLST). Traditionally, MLST is used to type isolated bacterial strains based on their allelic sequence profiles of multiple gene loci [7]. The method is low-throughput and is biased towards cultivatable strains. In a population composed of mixed strains from a species, sequenced alleles of various gene loci cannot be linked to specific strains, prohibiting the application of MLST on metagenomic shotgun sequencing data. The program MetaMLST [8] overcomes some of these challenges and is able to identify the most abundant sequence type profile for each species by reconstructing the locus sequences and cross-referencing the sequences with publicly available MLST databases. However, it does not provide a quantitative assessment of the relative abundances of the strains identified.

In this study, we developed a metagenomic MLST method, named MG-MLST, which combines MLST with high-throughput sequencing and uses STRUCTURE, a program designed to use genotype data to infer population structure [9], to identify the strain composition of a microbial community. We validated this method by characterizing the *P. acnes* strain composition in simulated data and the data from clinical skin samples.

## 2. Materials and Methods

### 2.1. STRUCTURE Running Parameters

Unless otherwise noted, STRUCTURE (version 2.3.4, July 2012, Stanford University, Stanford, CA, USA) was run under the parameters described below [10,11]. Samples were allowed to have a “mixed ancestry” by running the Admixture model with correlated allele frequencies at a ploidy of 100 for each sample and examining the gene loci of interest based on the MLST scheme being studied. STRUCTURE was run with a K = 6 or 10 based on the “learning sample” set of genomes included in the analysis. The value of K was selected to best represent the “real” biological clusters in the “learning sample” set of genomes, which had been determined based on the phylogenetic tree shown by Tomida et al. [12]. Each run was accompanied by a “learning sample” set of genomes with allelic profiles that were pre-defined to come from a specific cluster (ribotype (RT) group). This information was utilized by the USEPOPINFO feature of STRUCTURE (“Update allele frequencies using only individuals with POPFLAG=1” option turned on) to train the program to associate specific alleles with corresponding population groups. To supervise the convergence of the Markov chain to the defined population clusters for the “learning sample” set, the “Initialize at POPINFO” feature was turned on. Runs were ignored if the “learning sample” set was not properly predicted in which > 20% of the genomes were not correctly assigned to its pre-defined population. STRUCTURE was run at least 20 times for each sample with a 25,000 burn-in followed by 125,000 iterations. All other parameters were kept as the default. After running STRUCTURE, population groups that came from a single RT were combined, such as RT1 (clade IA-1 & clade IB-3 groups), for further analysis and comparison to 16S rRNA sequencing results.

### 2.2. Selecting Representatives of Population Groups for the “Learning Sample” Set

To determine which *P. acnes* genomes should be used as “learning samples” to represent the six population RT groups, we compared the MLST allelic profiles of the 82 genomes analyzed by Tomida et al. [12]. By comparing the allelic profiles of a single population group based on the gene loci from the Aarhus scheme (*cel*, *coa*, *fba*, *gms*, *lac*, *pak*, *oxc*, *recA*, and *zno*) [3], a consensus allelic profile of the nine genes was obtained. Strains that had an ambiguous allelic profile resembling multiple RT groups were removed. Strains that contained an allele not included in the list of alleles given by the Aarhus scheme data set were also removed. In addition, strains from rare RTs (not the top 10 most abundant RTs) described in Fitz-Gibbon et al. [5] or those in clade IC were removed. A total of 62 strains were chosen for the “learning sample” set for the Aarhus scheme (Table S1).

The “learning sample” set for the Belfast MLST_4_ scheme, which consisted of 64 strains (Table S1), was built using the same criteria as mentioned above.

When building the “learning sample” set for the combined Aarhus–Belfast scheme, an additional five genomes with unique allelic profiles were included due to the increased resolution provided by the combined set of eight genes. These genomes were divided into four new population groups based on their allelic profiles: an SK–RT1 group to represent RT1 strains that are similar to RT4/5, an HL025PA1–RT1 group to represent RT1 strains that resemble strain HL025PA1, a TIC group to represent a unique RT4/5 allelic profile, and an SK187-RT3 group to represent a unique RT3 allelic profile (Table S1). One genome, HL106PA1, had to be removed, because it had a novel allele for one of the Belfast MLST_4_ genes. In total, the “learning sample” set for the Aarhus–Belfast scheme consisted of 66 genomes that can be divided into 10 population groups.

### 2.3. Building Simulated Microbial Communities

Simulated microbiomes were generated by randomly selecting *P. acnes* genomes from the “learning sample” set to make up the designed relative abundance of each RT group in that simulated community. Microbiomes were all constructed to have 100 members to ease the transition from relative abundance of alleles to copies of the allele input into STRUCTURE. When rounding the relative abundance of an allele to an integer, occasionally, a community would be predicted to have either 99 members or 101 members. In these cases, either a missing value was inserted to fill the community to 100 members, or one fewer member was chosen from the RT group with the highest relative abundance to limit the community to 100 members.

For Set A simulated microbiomes, each was composed of only a single RT group, and therefore only strains with the designated RT would be selected from the “learning sample” set.

For Set B simulated microbiomes, the relative abundance of each RT group was randomly generated. The strains in each microbiome were randomly selected from the corresponding RT group in the “learning sample” set to reach to the relative abundance generated for that specific RT group.

For Set C simulated microbiomes, the relative abundance of each RT group (RT1/7/9, RT2/6, RT3, RT4/5/10, RT8) was set based on the samples analyzed in the previous 16S rRNA sequencing study of the skin microbiome [5]. The strains in each microbiome were randomly selected from the corresponding RT group in the “learning sample” set to reach to the relative abundance set for that specific RT group.

### 2.4. Sample Preparation and 454 Sequencing

Metagenomic DNA of six samples were previously obtained as described by Fitz-Gibbon et al. [5]. These samples came from four acne patients and two individuals with healthy skin. Four housekeeping genes (*fba*, *lac*, *zno*, and *recA*) were chosen for MLST based on Lomholt et al. [3]. This specific scheme was chosen due to its availability at the time of the experiments. Primers were designed as recommended by the Roche Genome Sequencer FLX System Technical Bulletin (454 Sequencing Technical Bulletin No. 013-2009) and included the Titanium Fusion Primer sequence, the key sequence, a MID tag, and a template-specific primer for each of the four gene loci. The template-specific primers were designed as documented in Lomholt et al. [3] with the exception of *recA*. The template-specific primer sequences are: *fba_F*, 5′-AGGACCCGCTATTCAACTCTCA-3′; *fba_R*, 5′-ACGCGGGTCGTACATCTTCTT-3′; *lac_F*, 5′-GCCGCAGCCTTGGGACTCT-3′; *lac_R*, 5′-GAAATGCTGTCGCCCCGTG-3′; *zno_F*, 5′-CGCCGGCATCACCACCTATT-3′; *zno_R*, 5′-TCTCACATCGCCCGCAACC-3′; *recA_F*, 5′-GCTTCCTCATACCACTGGTCATC-3′; and *recA_R*, 5′-CCGGAGACAACGACAGGT-3′. The metagenomic samples were assigned to a specific MID tag for identification purposes. The metagenomic DNA from each sample was then amplified using primers with its assigned MID tag following the Platinum High Fidelity Taq DNA polymerase protocol (Invitrogen, Carlsbad, CA, USA) in four multiplex PCR reactions. PCR conditions for each reaction were as follows: initial denaturation (95 °C, 3 min), 35 cycles of denaturation (94 °C, 30 s), extension (55 °C, 90 s), and elongation (72 °C, 90 s), and final extension (72 °C, 10 min). Amplification was verified by agarose gel electrophoresis. Multiplex reaction products were then purified with either one of the two methods: (1) Gel purification using the ZymoClean Gel DNA Recovery Kit (Zymo Research, Irvine, CA, USA) or (2) PCR clean-up using the DNA Clean & Concentrator Kit (Zymo Research, Irvine, CA, USA). Concentrations were determined by Nanodrop 1000 (Thermo Fisher Scientific, Waltham, MA, USA). The four multiplex reactions for each sample were normalized to the concentration of the least amplified locus. Samples were then pooled and sequenced with the Roche pyrosequencing platform (Roche, Branford, CT, USA). Sequencing reads were demultiplexed and then cleaned using PRINSEQ with the requirement of a quality score higher than 20. The clean reads were then mapped against the complete *P. acnes* genome HL096PA1 [13] with at least 80% identity. Each amplicon region examined had an average coverage of at least 12×.

### 2.5. Metagenomic Shotgun MLST Data

Metagenomic shotgun sequencing data for 26 clinical skin samples were obtained using Illumina HiSeq platform as previously reported by Barnard et al. [14]. Low quality reads and human reads were filtered out first. Reads of marker genes were extracted by mapping the cleaned reads against the *P. acnes* genome HL096PA1 [13] with at least 80% identity using Bowtie2 [15]. All samples had an average coverage on the *P. acnes* genome of at least 25×.

### 2.6. Building Microbiome Allelic Profiles

To build a microbiome allelic profile, a comparison was first performed using the available *P. acnes* alleles listed on https://pubmlst.org/ and previously on www.mlst.net to determine which single nucleotide polymorphisms (SNPs) distinguished each allele and would act as markers for the absence/presence of an allele. Alleles that were not found in the “learning sample” set were ignored. A list of marker SNPs for each allele used for the analysis can be found in Table S4.

The sequence coverage at each position of the marker genes (*fba*, *lac*, *recA*, *zno*, *aroE*, *guaA*, *tly*, and *camp2*) was used to calculate the relative abundance of each marker SNP in the samples. For a marker SNP to be considered present, it needs to meet the criteria similar to those adopted by Schloissnig et al. [6]: (1) the SNP has to have a relative abundance of at least 1%, and (2) the SNP needs to be supported by at least four reads. The marker SNPs were then used to derive the strain allelic profile of the sample based on a second set of criteria: (1) all marker SNPs for an allele must be present for that allele to be considered present, and (2) all other alleles not covered by the marker SNPs were considered to be an RT1 allele (*fba* 2, *lac* 4, *recA* 5, *zno* 6, *aroE* 1, *guaA* 3, *tly* 1, and *camp2* 1).

To simplify the conversion of the relative abundance of alleles to a strain allelic profile, the allelic profiles input into STRUCTURE used a ploidy of 100 for each allele, so that if an allele had a relative abundance of 10%, 10 copies of that allele were put into the allelic profile for the microbiome.

### 2.7. MetaMLST Analysis

To compare MG-MLST with MetaMLST, we constructed simulated metagenome data to test MetaMLST. We randomly sampled 1 Mbp sequences from the sequencing data of *P. acnes* genomes. In each simulated metagenome, two *P. acnes* genomes of different ribotypes were selected to create microbiome compositions with varying ratios between the two strains (0.8 Mbp/0.2 Mbp, 0.7 Mbp/0.3 Mbp, 0.6 Mbp/0.4 Mbp, and 0.5 Mbp/0.5 Mbp). Each simulated composition was tested in 10 randomly generated trials. These 10 sets of metagenome data were then analyzed through MetaMLST. The output, which is the most dominant sequence type of the species in the microbiome, was then cross-referenced to determine the corresponding ribotype.

### 2.8. Statistical Analysis

For all data sets, 20 runs in which the “learning sample” set samples clustered into their supervised populations were selected for statistical analysis.

## 3. Results

### 3.1. Combination of MLST and STRUCTURE for Strain Identification and Quantification

In this study we investigated whether we can utilize the program STRUCTURE to identify strain populations and quantify their relative abundances from microbiome data, which we named as the MG-MLST method. The program STRUCTURE was designed to use genotype data to infer population structure and is capable of determining an individual’s ancestry, population membership, and migrant status [9]. Since its release, STRUCTURE has been used to study population genetics in a variety of microbial organisms [10,16,17]. One specific study applied it to predict the ancestry of multiple *H. pylori* isolates using individually sequenced MLST alleles [18]. This prompted us to consider whether STRUCTURE can also be applied to microbial community samples to predict the strain population. We hypothesized that by treating a metagenomic sample as if it was a polyploidy admixed individual that inherits its MLST alleles from a set of predefined “ancestral” cluster populations, we can use STRUCTURE’s “ancestry” prediction as a representative of the percent relative abundance of these clusters in the sample. With our clusters acting as representatives of the various strain populations, we can then infer strain-level composition, thereby applying MLST on metagenomic samples.

### 3.2. Selection of “Learning Sample” Set

We selected *P. acnes* as a test species for our method MG-MLST, because it is well studied at the strain level with multiple MLST typing schemes and 16S ribotyping available as well as over 100 sequenced genomes. *P. acnes* is a dominate species on human skin and has been implicated to play roles in both skin health and disease. *P. acnes* strains have been classified using a variety of marker gene-based typing methods, including several MLST schemes mainly based on the Belfast and Aarhus schemes [3,4,19,20,21], single locus sequence typing (SLST) [22], and 16S rRNA ribotyping [5]. Based on a large number of sequenced genomes, *P. acnes* strains are clustered into eight phylogenetic clades, which correspond to specific 16S ribotypes and clonal complexes (CC) based on the MLST schemes [12].

In order to use the ancestry prediction algorithm in STRUCTURE [9] to determine the strain populations in a microbial community, we first selected a set of “learning samples”. The “learning samples” represent the possible population groups that the test samples may have originated from and contain genetic markers that best represent the strain-level population groups within the species. For *P. acnes,* we considered six major strain population groups found on human skin, which are RT1 representing clade IA-1, RT2/6 representing clade II, RT3 representing clade IB-2, RT4/5 representing clade IA-2, RT8 representing clade IB-1, and RT1/IB-3 representing clade IB-3. Clades IC and III were not considered because they are rarely found on facial skin and only a couple of genome sequences are currently available. A total of 62 strains representing the six major strain population groups were chosen as the “learning sample” set, which consisted of 15 RT1 strains, 11 RT2/6 strains, 16 RT3 strains, 12 RT4/5 strains, six RT8 strains, and two RT1 (clade IB-3) strains. All the genomes of these strains are available. A list of the strains and their corresponding groups is described in Table S1.

The MG-MLST method relies on the allelic sequences of the genetic loci used in MLST as the genetic markers for identifying the strain population groups. For *P. acnes,* we used the MLST allelic profiles of the sequenced strains described by Tomida et al. [12]. Both Belfast and Aarhus MLST schemes were examined.

### 3.3. Using STRUCTURE to Determine Strain-Level Composition on Simulated Microbiome Data

To determine whether the program STRUCTURE can accurately predict the strain population structure of the microbiome, we tested the program using the simulated microbiome of varying compositions of the *P. acnes* strains included in the “learning sample” set (details in Methods). The allelic profiles of the MLST loci in each simulated microbiome were constructed at various relative abundances, and STRUCTURE was used to predict the strain composition based on the overall allelic profile.

To enhance the computational efficiency of the prediction by STRUCTURE, we first identified the key genetic loci in the Aarhus MLST scheme. The Aarhus scheme uses nine genetic loci to classify *P. acnes* strains [3]. By constructing phylogenetic trees using various combinations of the subgroups of the nine genes and comparing them to the phylogenetic tree constructed based on all nine genes, we identified four genes (*fba*, *lac*, *recA*, and *zno*) that best separated the six population groups. To validate this four-gene scheme, we compared the STRUCTURE results for the simulated microbiomes to the results obtained from the nine-gene scheme. The predictions using the two schemes highly correlated with a Pearson’s correlation value of 1 (Figure S1), suggesting that the additional five genes of the Aarhus scheme are not essential in distinguishing the six major clades. Henceforth, all further tests were performed using the Aarhus four-gene set.

**Figure 1:**
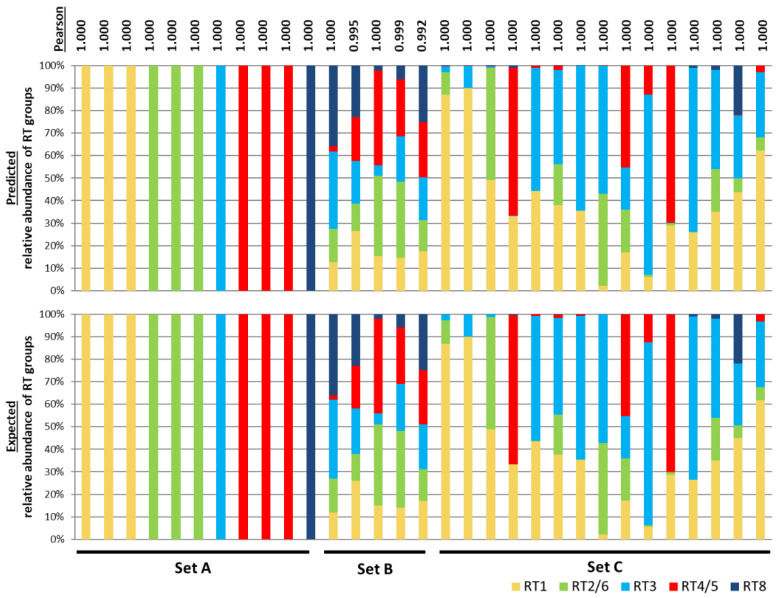
The strain composition predicted by STRUCTURE is highly consistent with the expected *P. acnes* populations based on simulated data. The top panel shows the ribotype (RT) group composition predicted by STRUCTURE based on the simulated communities. The bottom panel shows the expected RT group composition. Each column represents the relative abundances of the RT groups in each sample. The expected composition of each simulated community is listed in Table S2. Set A communities were generated to contain a single RT group per sample. Set B communities were generated to randomly have varying relative abundances of the RT groups. Five representative samples among the total 100 simulated communities are shown. Figure S2 lists all 100 communities of Set B. Set C communities were generated to mimic the population structures from previously characterized clinical skin samples [5]. Pearson’s correlations were calculated to compare the predicted population composition with the expected data as shown on the top.

Three types of simulated microbiomes, Set A, Set B, and Set C, were generated to represent various community compositions observed in the skin microbiome associated with acne (Table S2). Set A represented cases in which only one or two closely-related strains dominate the population. These populations were built with only one or two *P. acnes* RT groups. The possible strain compositions are RT1 (clade IA-1), RT1 (clade IB-3), RT2, RT3, RT4, RT5, RT6, RT8, RT2&6, RT4&5, or RT1 (clade IA-1 & clade IB-3) strains. In Set A, STRUCTURE accurately predicted the strain compositions with a Pearson’s correlation of 1 between observed and expected populations (Figure 1).

Set B represented populations with varying relative abundances of multiple RT groups that were randomly generated. The predictions of 100 populations of Set B by STRUCTURE all highly correlated with the expected results, with a Pearson’s correlation of 0.992–1.000 (Figure S2). Five representative communities from Set B are shown in Figure 1.

Set C microbiomes mimic the RT compositions found in clinical samples from aprevious study [5]. As shown in Figure 1, although the communities in Set C were highly variable in composition, all predictions highly correlated with their expected compositions with a Pearson’s correlation of 1.

These results based on the simulated data demonstrated the validity of using STRUCTURE to predict the strain composition of a microbiome.

### 3.4. Using MG-MLST to Determine Strain-Level Composition in MLST Amplicon Sequencing Data of Clinical Samples

**Figure 2:**
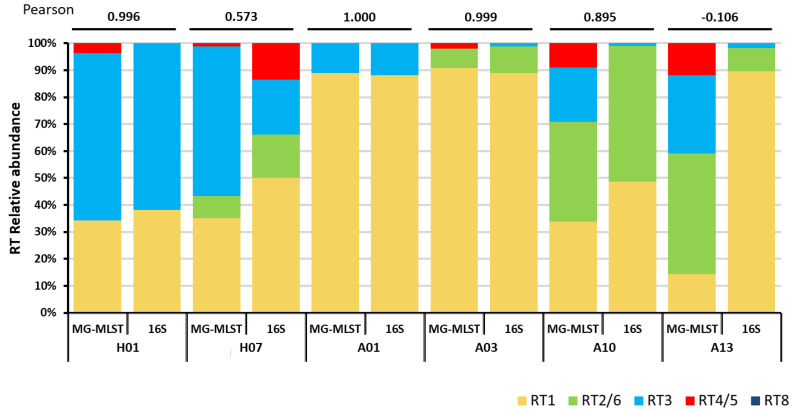
The strain composition predicted by metagenomic multi-locus sequence typing (MG-MLST) is highly consistent with the *P. acnes* population structure based on 16S ribotyping. The first column of each sample represents the predicted strain composition using the sequence data obtained from the 454 amplicon library. The second column represents the strain composition based on the 16S ribotype data [5]. Pearson’s correlations between the two methods are shown on the top.

We next tested whether the MG-MLST method can be applied to identify the strain populations of the microbiome in clinical samples, in which the MLST marker genes were amplified and sequenced using high-throughput sequencing. We prepared amplicon libraries from six clinical samples, which were previously classified at the strain level using 16S ribotyping [5]. The four genes of the Aarhus scheme (*fba*, *lac*, *recA*, and *zno*) were amplified, and the amplicon libraries were sequenced using 454 pyrosequencing platform to obtain longer reads. Reads of an average length of 472 bp were mapped to the four genes and used to determine the relative abundances of marker SNPs for each allele. The relative abundance of each allele was then inferred from the marker SNPs present and an allelic profile of the *P. acnes* strain population was generated and input into STRUCTURE (Methods). The “learning sample” set described earlier was used for strain population prediction. When compared to the previous 16S ribotyping results [5], four of the six samples had a strong correlation, with a Pearson’s correlation of at least 0.895 (Figure 2). The other two samples, H07 and A13, were predicted to have much lower RT1 abundances than previously reported by 16S ribotyping. This difference may be due to the greater genomic variation among RT1 strains, some of which have similar allelic profiles to other RTs including RT3, RT4, and RT5 [12]. Overall, this result suggests that the MG-MLST method can infer the strain population based on the MLST amplicon sequence data.

### 3.5. Using MG-MLST to Determine Strain-Level Composition in Metagenomic Shotgun Sequencing Data of Clinical Samples

Because amplification of genetic loci used in MLST amplicon sequencing may introduce biases toward certain alleles, metagenomic shotgun sequencing data can be a better alternative for MG-MLST. Given that more microbiome studies are utilizing metagenomic shotgun sequencing analysis, we determined whether MG-MLST can be applied to this type of data. We obtained the metagenomic shotgun sequencing data of 26 skin samples [14]. These samples were previously analyzed at the strain level using 16S ribotyping. Sequence reads mapped to the four Aarhus genes (*fba*, *lac*, *recA*, and *zno*) were extracted with an identity threshold of 80%. The alleles of each gene were identified based on the marker SNPs. We then generated an allelic profile of the relative abundance of each allele of all four genes present in each sample. This allelic profile was input into STRUCTURE to predict the strain composition.

**Figure 3:**
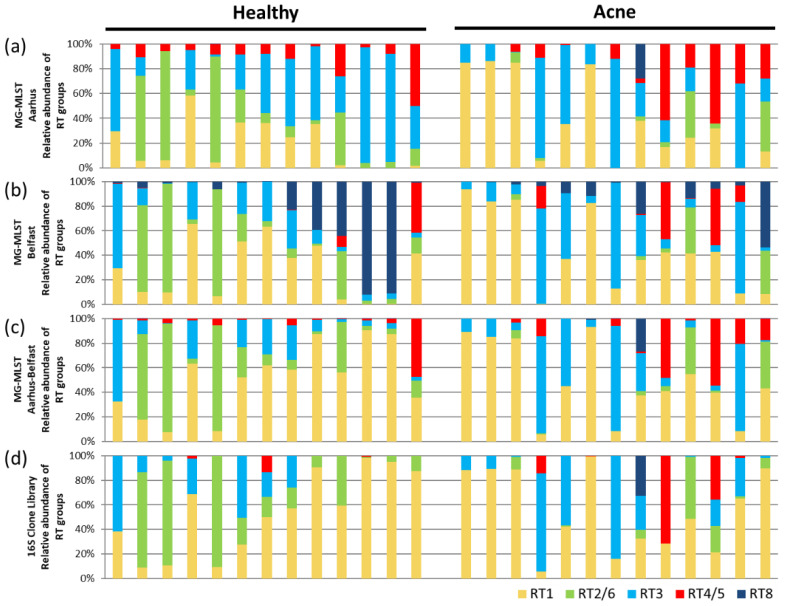
Comparison of the MLST schemes in predicting strain composition using MG-MLST. (**a**) Ribotype (RT) group composition predicted by STRUCTURE based on the Aahrus four gene set (*fba*, *lac*, *recA*, and *zno*). (**b**) RT group composition predicted by STRUCTURE based on the Belfast MLST_4_ scheme (*aroE*, *guaA*, *tly*, and *camp2*). (**c**) RT group composition predicted by STRUCTURE based on the combined eight gene set (Aarhus–Belfast) (*fba*, *lac*, *recA*, *zno*, *aroE*, *guaA*, *tly*, and *camp2*). (**d**) RT group composition based on 16S ribotype data. Pearson’s correlations are shown in Table S3.

When the strain compositions of the 26 samples predicted by MG-MLST were compared to the 16S ribotyping, 65% of the predicted populations (17 samples) strongly correlated, with a Pearson’s correlation ≥ 0.74 (Figure 3a, Table S3). Two samples (8%) had moderate correlations of 0.67 and 0.48, respectively. The other seven samples did not correlate well. We observed that MG-MLST predictions based on the four genes of the Aarhus scheme tended to overestimate the presence of the RT4/5 strain group (Figure 3a). This analysis suggests that MG-MLST can be used to determine the strain composition from metagenomic shotgun sequencing data, while the selection of the MLST marker genes can be improved.

To investigate whether the MG-MLST method can be improved by using a different set of genetic loci, we considered the four genes of the Belfast MLST_4_ scheme (*aroE*, *guaA*, *tly*, and *camp2*) [20]. We re-ran MG-MLST predictions on the metagenomic shotgun data of the 26 clinical samples described above. A similar outcome was obtained. A total of 62% of the predicted populations (16 samples) strongly correlated with the 16S ribotyping with a Pearson’s correlation ≥ 0.73 (Figure 3b, Table S3). Most of the samples that had medium to low correlations based on the Aarhus gene set also weakly correlated using the Belfast MLST_4_ scheme. However, unlike the Aarhus scheme, the Belfast MLST_4_ scheme did not overestimate the abundance of the RT4/5 group, but rather often overestimated the presence of the RT8 group, resulting in a lower abundance of the RT3 group. The overestimation of certain RT groups in one scheme but not in another is likely due to the chosen gene set, as the resolution provided by only four “informative” MLST genes might be limited. We, therefore, hypothesized that by combining the genes from both schemes the resolution may be improved.

To test this hypothesis, we combined the genes of the Aarhus and Belfast MLST_4_ schemes to create a new MLST scheme consisting of eight informative genes (*fba*, *lac*, *recA*, *zno*, *aroE*, *guaA*, *tly*, and *camp2*). With the increased resolution provided by the combined gene set, we added five additional *P. acnes* genomes with unique allelic profiles to the “learning sample” set to account for less common strains that may exist in the community. The combination of these two MLST schemes largely improved the MG-MLST prediction. The number of samples with high correlations increased to 23 (88%, Pearson’s correlation > 0.71) (Figure 3c, Table S3). Furthermore, all samples that had negative correlations previously were improved except for one sample with a Pearson’s correlation of 0.24. These results suggest that with additional genetic information from a more “informative” set of gene loci with a more diverse “learning sample” set, a clearer picture of the strain population with relative abundances can be obtained from MG-MLST.

### 3.6. Using MG-MLST to Study Strain-Level Differences in the Skin Microbiome between Acne Patients and Healthy Individuals

To demonstrate the application of the MG-MLST method in identifying strain-level differences in the microbiome and their associations with health and disease, we applied MG-MLST to our study of the skin microbiome in acne. Among the 26 samples used in the analyses mentioned above, 13 were from healthy individuals and 13 from acne patients. Three MLST gene sets: the Aarhus four gene set, the Belfast MLST_4_ gene set, and the combined eight gene set from both schemes (Aarhus–Belfast), were used in MG-MLST to detect strain-level differences between the acne group and healthy group. Regardless of the gene set used, RT2/6 was consistently different in relative abundance between acne and healthy cohorts (*p* = 0.059–0.063). This finding is consistent with the previous studies, which have shown that RT2 and RT6 are health-associated strains [5,14]. All other RT groups showed little difference between acne patients and healthy individuals in relative abundance, possibly due to the relatively small sample size compared to previous studies [5,14]. The differences detected by the MG-MLST method based on the combined eight gene set (Aarhus–Belfast) were similar to those observed by the 16S rRNA clone library analysis, demonstrating the ability of the method to detect strain-level differences in the microbial communities between health and disease.

### 3.7. Comparison between MG-MLST and MetaMLST

We compared our method to MetaMLST, a program that is also designed to analyze the microbiome at the strain level [8]. MetaMLST uses metagenomic shotgun sequencing data to reconstruct the gene loci of the most abundant sequence type of a particular species and match it against the MLST database, PubMLST (pubmlst.org). We applied the sequencing data from the same 26 clinical samples mentioned above to MetaMLST. MetaMLST was able to identify the top dominant sequence type in 13 of the 26 samples. Of these 13 samples, the identified sequence types for 11 samples could be cross-referenced to known strains and RTs, while the sequence types for the remaining two samples did not match to known RTs and were assigned as new (Table 1).

Since MetaMLST outputs only the most dominant sequence type present in the microbiome data with no relative abundance, to compare the results of MG-MLST to those of MetaMLST, we compared the most abundant *P. acnes* strain determined by MG-MLST to the sequence type determined by MetaMLST. Among the 11 samples that yielded a known sequence type by MetaMLST, the assigned strains were consistent with the most abundant RT determined by MG-MLST except for one discrepancy between the two methods. For sample H09, MG-MLST identified that the most abundant ribotype was RT1, which is consistent with the result based on 16S ribotyping, while MetaMLST identified the RT8 strain as the most dominant (Table 1). Overall, MG-MLST and MetaMLST are comparable in the identification of the most dominant strain in the population, while MG-MLST provides the composition of other less abundant strains from the same species as well as quantifies the relative abundance of each strain in the microbiome.

## 4. Discussion

It is becoming increasingly recognized that understanding strain-level differences in the microbiome is important. Certain strains of a species may contain specific genes that contribute to the functional differences in the microbial community. Strain-level differences in the microbiome may reveal new associations between the microbial community and health or disease that were previously overlooked at higher taxonomic levels [23].

Many recent microbiome studies have employed metagenomic shotgun sequencing instead of 16S rRNA amplicon sequencing in order to improve the resolution in characterizing the taxonomic and functional composition of the microbiome. This trend has been accompanied with an increasing demand in tools that can utilize metagenomic reads to characterize the microbial community at the species and strain levels. Although computationally intensive, tools like Sigma [24] and Pathoscope [25] were developed to analyze the microbiome at the strain level by mapping metagenomic reads to reference genomes and infer strain population structure based on mapped reads. For organisms with only a few genomes sequenced, it is challenging to use these mapping-based algorithms as they may not be able to fully reveal the diversity of the organism at the strain level. Alternatively, tools like MetaPhlAn can determine the microbiome at the strain level by using clade-specific marker genes as references instead of whole genomes [26], but it still requires a large reference set of marker genes.

The MG-MLST method presented here has several unique advantages in analyzing the metagenome at the strain level. First, MG-MLST does not require a large set of reference genomes to assign reads, rather, the method is dependent on the allelic profiles of strains. This makes MG-MLST more affordable for organisms whose genomes have not been extensively sequenced, since only a few sequences of housekeeping genes from reference strains are needed. Additionally, the MLST sequence profiles of hundreds of strains of hundreds of species are available in public databases, such as PubMLST, and can be readily used as references. Second, MG-MLST can be used to analyze both metagenomic shotgun data and marker gene amplicon data. In the cases where the strain populations of only a specific organism are investigated, sequence data from amplicon libraries can be used, which could be more cost effective. Compared to MetaMLST, which is similar in data requirement but identifies only the most dominant strain of a species without assigning the relative abundance, MG-MLST provides a quantitative analysis of the composition of the strain populations in the microbiome with diversity and richness.

While MG-MLST can be used to quantitatively analyze microbial communities at the strain level, the method has limitations and can be further developed. Because it utilizes MLST data, while the method performs well when applied to the bacterial species with well characterized MLST schemes, it is limited in ability to interrogate the species with limited or no MLST data. Additionally, common to all reference-based methods, a major obstacle is the method’s ability to identify new strain types. The method analyzes the microbiome based on known allelic profiles of the strains of a given species. If new allelic profiles in the microbiome data are not included in the “learning sample” set, the present algorithm will artificially assign the alleles to one of the known populations rather than defining a new population group.

Another important factor to consider when performing MG-MLST is the selection of the “learning sample” set and the marker genes used to distinguish population groups. As shown in Figure 3, these two factors can influence the predicted strain composition. By using more informative MLST gene markers and including reference strains with more diverse allelic profiles, our identification of the strains and their relative abundances by STRUCTURE was improved. This increased accuracy is often accompanied by increased computational complexity, therefore, a balance must be considered based on the biological questions being asked.

## 5. Conclusions

To enable investigations of the microbiome differences at the strain level, in this study we developed MG-MLST, a method that combines MLST and high-throughput sequencing and uses the “ancestry prediction” algorithm of STRUCTURE to quantitatively determine the strain-level composition of a microbial community. With the proper selection of a few MLST marker genes, one can perform the analysis without the requirement of a large set of reference genomes. MLST allelic profiles of strains can be generated in-house or obtained from public databases, making this method more cost-effective for organisms that do not have genomes of multiple strains available. As demonstrated in this study, the MG-MLST method can be applied to clinical studies to investigate differences in the microbiome at the strain-level between healthy and diseased states.

## Figures and Tables

**Table 1:** Comparison of top strain assignment between MG-MLST and MetaMLST.

Sample	MG-MLST			MetaMLST	
	Sequence Type (ST) Assigned	Ribotype (RT) Assigned	Relative Abundance of RT	Sequence Type (ST) Assigned	Ribotype (RT) Assigned
H01	6,7,25,27,28,30	2/6	0.869	7	6
H02	6,7,25,27,28,30	2/6	0.704	30	2
H03	6,7,25,27,28,30	2/6	0.886	100	New
H04	1,5	1	0.656	New	New
H05	2	3	0.691	2	3
H06	1,5	1	0.511	5	1
H07	1,5	1	0.634	New	New
H08	1,5	1 3	0.378 0.316	New	New
H09	1,5	1 8	0.477 0.395	4	8
H10	4,13,21	8 2/6	0.443 0.393	New	New
H11	4,13,21	8	0.922	4	8
H12	4,13,21	8	0.912	4	8
H13	1,5	1 4/5	0.414 0.409	115	1
A01	1,5	1	0.935	1	1
A02	1,5	1	0.840	New	New
A03	1,5	1	0.851	53	New
A04	2,22,23,24,36,91	3	0.779	2	3
A05	2	3	0.537	New	New
A06	1,5	1	0.825	New	New
A07	2	3	0.862	New	New
A08	6,7,25,27,28,30	1	0.360	New	New
A09	3,10,11,17,70	4/5 1	0.468 0.423	New	New
A10	1,5	1 2/6	0.414 0.377	New	New
A11	3,10,11,17,70	4/5 1	0.460 0.426	New	New
A12	2,22,23,24,36,91	3	0.747	22	3
A13	4,13,21	8	0.538	New	New

Samples highlighted in gray have consistent assignment between the two methods.

## Supplementary Materials

The following are available online at https://www.mdpi.com/2076-2607/8/5/684/s1.
