# Supplementary material for: MG-MLST: Characterizing the Microbiome at the Strain Level in Metagenomic Data
Source: Microorganisms. 2020 May 8;8(5):684. doi: 10.3390/microorganisms8050684 (PMC7284976; doi:10.3390/microorganisms8050684)
Supplement: Supplementary file 1 [file microorganisms-08-00684-s001.zip › MLSTPaper-SupplementaryFigureS1.pdf]

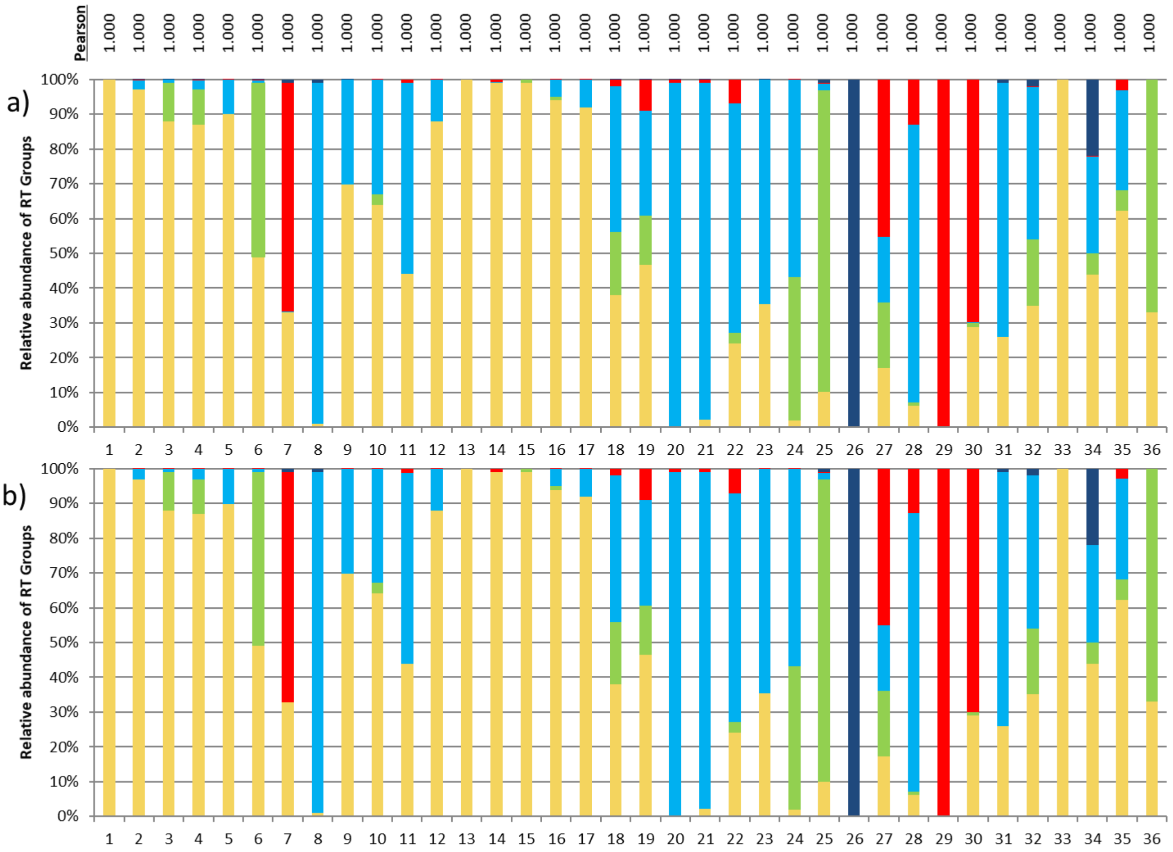

Figure S1. Comparison of the predicted strain compositions by STRUCTURE based on (a) 4 (*fba, lac, recA, zno*) or (b) 9 (*cel, coa, fba, gms, lac, oxc, pak, recA, zno*) Aarhus MLST gene loci.
