# Supplementary material for: MG-MLST: Characterizing the Microbiome at the Strain Level in Metagenomic Data
Source: Microorganisms. 2020 May 8;8(5):684. doi: 10.3390/microorganisms8050684 (PMC7284976; doi:10.3390/microorganisms8050684)
Supplement: Supplementary file 1 [file microorganisms-08-00684-s001.zip › MLSTPaper-SupplementaryTableS1_final.pdf]

Table S1. ***P. acnes*** **strains included in the "Learning Sample" set.**

Strain	Aarhus	Belfast	Aarhus-Belfast
266	RT1	RT1	RT1
6609	Not Included	TIB3	Not Included
ATCC11828	RT2/6	RT2/6	RT2/6
HL074PA1	Not Included	RT4/5	Not Included
HL001PA1	RT2/6	RT2/6	RT2/6
HL002PA1	Not Included	RT3	Not Included
HL002PA2	RT1	RT1	RT1
HL002PA3	RT1	RT1	RT1
HL005PA1	RT4/5	RT4/5	RT4/5
HL005PA2	RT1	RT1	RT1
HL005PA3	RT1	RT1	RT1
HL005PA4	RT3	RT3	RT3
HL007PA1	RT4/5	RT4/5	RT4/5
HL013PA1	Not Included	RT3	Not Included
HL013PA2	RT1	RT1	RT1
HL020PA1	RT1	RT1	RT1
HL025PA1	Not Included	Not Included	HL025PA1-RT1
HL025PA2	RT3	RT3	RT3
HL027PA1	RT3	RT3	RT3
HL027PA2	RT1	RT1	RT1
HL030PA1	TIB3	TIB3	TIB3
HL030PA2	RT3	RT3	RT3
HL036PA3	RT1	RT1	RT1
HL037PA1	RT3	RT3	RT3
HL038PA1	RT4/5	RT4/5	RT4/5
HL042PA3	RT2/6	RT2/6	RT2/6
HL043PA1	RT4/5	RT4/5	RT4/5
HL043PA2	RT4/5	RT4/5	RT4/5
HL045PA1	RT4/5	RT4/5	RT4/5
HL046PA1	RT3	RT3	RT3
HL046PA2	RT1	RT1	RT1
HL050PA1	RT3	RT3	RT3
HL050PA3	RT3	RT3	RT3
HL053PA1	RT4/5	RT4/5	RT4/5
HL053PA2	RT8	RT8	RT8
HL056PA1	RT4/5	RT4/5	RT4/5
HL060PA1	RT2/6	RT2/6	RT2/6
HL063PA1	RT1	RT1	RT1
HL063PA2	RT3	RT3	RT3
HL067PA1	RT3	RT3	RT3
HL074PA1	RT4/5	Not Included	RT4/5
HL082PA1	RT8	RT8	RT8
HL082PA2	RT2/6	RT2/6	RT2/6
HL083PA2	RT3	RT3	RT3
HL086PA1	RT8	RT8	RT8
HL087PA1	RT3	RT3	RT3
HL087PA2	RT1	RT1	RT1
HL087PA3	RT3	RT3	RT3
HL092PA1	RT8	RT8	RT8
HL096PA1	RT4/5	RT4/5	RT4/5
HL096PA2	RT4/5	RT4/5	RT4/5
HL096PA3	RT1	RT1	RT1
HL097PA1	Not Included	Not Included	TIC

HL099PA1	RT4/5	RT4/5	RT4/5
HL100PA1	RT1	RT1	RT1
HL103PA1	RT2/6	RT2/6	RT2/6
HL106PA1	RT2/6	Not Included	Not Included
HL110PA1	RT8	RT8	RT8
HL110PA2	RT8	RT8	RT8
HL110PA3	RT2/6	RT2/6	RT2/6
HL110PA4	RT2/6	RT2/6	RT2/6
HL202PA1	RT2/6	RT2/6	RT2/6
J139	RT2/6	RT2/6	RT2/6
J165	RT1	RT1	RT1
KPA171202	TIB3	TIB3	TIB3
P.acn17	RT3	RT3	RT3
P.acn31	RT3	RT3	RT3
P.acn33	Not Included	RT3	Not Included
PRP-38	Not Included	Not Included	TIC
SK137	Not Included	Not Included	SK-RT1
SK182	Not Included	Not Included	SK-RT1
SK187	RT3	Not Included	SK187-RT3

*Strains not included in the “Learning Sample” set for a particular MLST scheme are labeled "Not Included".
