# Supplementary material for: MG-MLST: Characterizing the Microbiome at the Strain Level in Metagenomic Data
Source: Microorganisms. 2020 May 8;8(5):684. doi: 10.3390/microorganisms8050684 (PMC7284976; doi:10.3390/microorganisms8050684)
Supplement: Supplementary file 1 [file microorganisms-08-00684-s001.zip › MLSTPaper-SupplementaryTableS2_final.pdf]

Table S2. Expected RT group composition of each simulated comminity.

		Relative abundance of each RT group				
Set	Community	RT1	RT2/6	RT3	RT4/5	RT8
A	A1	1	0	0	0	0
A	A2	1	0	0	0	0
A	A3	1	0	0	0	0
A	A4	0	1	0	0	0
A	A5	0	1	0	0	0
A	A6	0	1	0	0	0
A	A7	0	0	1	0	0
A	A8	0	0	0	1	0
A	A9	0	0	0	1	0
A	A10	0	0	0	1	0
A	A11	0	0	0	0	1
B	B1	0.12	0.15	0.35	0.02	0.36
B	B2	0.26	0.12	0.2	0.19	0.23
B	B3	0.11	0.3	0.15	0.23	0.21
B	B4	0.11	0.33	0.14	0.22	0.2
B	B5	0.29	0.26	0.05	0.21	0.19
B	B6	0.15	0.36	0.05	0.42	0.02
B	B7	0.14	0.34	0.21	0.25	0.06
B	B8	0.1	0.19	0.29	0.01	0.41
B	B9	0.04	0.15	0.32	0.22	0.27
B	B10	0.12	0.27	0.02	0.25	0.34
B	B11	0.23	0.33	0.26	0.06	0.12
B	B12	0.2	0.21	0.23	0.15	0.21
B	B13	0.14	0.24	0.3	0.23	0.09
B	B14	0.22	0.22	0.27	0.2	0.09
B	B15	0.27	0.25	0.18	0.09	0.21
B	B16	0.32	0.19	0.02	0.29	0.18
B	B17	0.26	0.21	0.28	0.12	0.13
B	B18	0.06	0.38	0.08	0.33	0.15
B	B19	0.1	0.29	0.24	0.16	0.21
B	B20	0.04	0.25	0.21	0.23	0.27
B	B21	0.26	0	0.04	0.33	0.37
B	B22	0.07	0.36	0.29	0	0.28
B	B23	0.21	0.15	0.17	0.25	0.22
B	B24	0.19	0.39	0.19	0.07	0.16
B	B25	0.17	0.47	0.03	0.3	0.03
B	B26	0.11	0.22	0.28	0.15	0.24
B	B27	0.41	0.34	0.19	0.05	0.01
B	B28	0.09	0.28	0.27	0.02	0.34
B	B29	0.33	0.08	0.22	0.1	0.27
B	B30	0.68	0.01	0.21	0.03	0.07
B	B31	0.12	0.19	0.15	0.27	0.27
B	B32	0.24	0.24	0.18	0.27	0.07
B	B33	0.2	0.26	0.32	0.08	0.14
B	B34	0.11	0.25	0.3	0.07	0.27
B	B35	0.1	0.16	0.4	0.04	0.3
B	B36	0.28	0.15	0.08	0.08	0.41
B	B37	0.03	0.26	0.27	0.13	0.31
B	B38	0.13	0.13	0.22	0.26	0.26
B	B39	0.15	0.09	0.42	0.11	0.23
B	B40	0.17	0.14	0.2	0.24	0.25
B	B41	0.21	0.22	0.27	0.17	0.13

B	B42	0.28	0.38	0.14	0.11	0.09
B	B43	0.29	0.24	0.13	0.05	0.29
B	B44	0.21	0.08	0.26	0.19	0.26
B	B45	0.22	0.15	0.04	0.25	0.34
B	B46	0.26	0.35	0.22	0.02	0.15
B	B47	0.28	0.16	0.3	0.11	0.15
B	B48	0.14	0.33	0.12	0.28	0.13
B	B49	0.17	0.12	0.29	0.26	0.16
B	B50	0.27	0.25	0.18	0.18	0.12
B	B51	0.18	0.23	0.17	0.22	0.2
B	B52	0.08	0.03	0.24	0.42	0.23
B	B53	0.05	0.36	0.09	0.24	0.26
B	B54	0.19	0.17	0.27	0.16	0.21
B	B55	0.18	0.04	0.21	0.31	0.26
B	B56	0.29	0.21	0.11	0.3	0.09
B	B57	0.07	0.27	0.1	0.26	0.3
B	B58	0	0.03	0.25	0.36	0.36
B	B59	0.21	0.38	0.11	0.15	0.15
B	B60	0.38	0.18	0.36	0.06	0.02
B	B61	0.08	0.17	0.05	0.42	0.28
B	B62	0.17	0.34	0.09	0.36	0.04
B	B63	0.09	0.29	0.35	0.12	0.15
B	B64	0.07	0.21	0.32	0.15	0.25
B	B65	0.17	0.09	0.23	0.3	0.21
B	B66	0.32	0.23	0.16	0.11	0.18
B	B67	0.13	0.16	0.32	0.24	0.15
B	B68	0.26	0.24	0.2	0.07	0.23
B	B69	0.12	0.22	0.25	0.24	0.17
B	B70	0.16	0.11	0.33	0.05	0.35
B	B71	0.22	0.01	0.4	0.23	0.14
B	B72	0.32	0.01	0.32	0.14	0.21
B	B73	0.28	0.28	0.03	0.19	0.22
B	B74	0.17	0.26	0.12	0.18	0.27
B	B75	0.03	0.22	0.31	0.22	0.22
B	B76	0.33	0.07	0.28	0.25	0.07
B	B77	0.25	0.09	0.17	0.27	0.22
B	B78	0.23	0.1	0.21	0.26	0.2
B	B79	0.26	0.2	0.25	0	0.29
B	B80	0.08	0.3	0.01	0.45	0.16
B	B81	0.36	0.17	0.04	0.27	0.16
B	B82	0.29	0.35	0.19	0.13	0.04
B	B83	0.1	0.26	0.23	0.32	0.09
B	B84	0.09	0.34	0.17	0.24	0.16
B	B85	0.14	0.28	0.18	0.07	0.33
B	B86	0.28	0.24	0.03	0.14	0.31
B	B87	0.19	0.26	0.14	0.25	0.16
B	B88	0.08	0.26	0.32	0.27	0.07
B	B89	0.5	0.2	0.01	0.23	0.06
B	B90	0.02	0.26	0.31	0.17	0.24
B	B91	0.27	0.09	0.19	0.27	0.18
B	B92	0.1	0.24	0.26	0.25	0.15
B	B93	0.2	0.3	0.07	0.1	0.33
B	B94	0.1	0.24	0.22	0.2	0.24
B	B95	0.23	0.29	0.22	0.03	0.23
B	B96	0.19	0.21	0.18	0.31	0.11
B	B97	0.2	0.15	0.15	0.23	0.27

B	B98	0.15	0.31	0.28	0.09	0.17
B	B99	0.27	0.17	0.23	0.05	0.28
B	B100	0.07	0.25	0.04	0.2	0.44
C	C1	0.868	0.104	0.028	0.000	0.000
C	C2	0.900	0.000	0.100	0.000	0.000
C	C3	0.488	0.499	0.013	0.000	0.000
C	C4	0.333	0.000	0.000	0.660	0.006
C	C5	0.435	0.003	0.554	0.008	0.000
C	C6	0.377	0.178	0.427	0.016	0.002
C	C7	0.352	0.004	0.637	0.004	0.004
C	C8	0.022	0.406	0.571	0.000	0.000
C	C9	0.172	0.188	0.188	0.453	0.000
C	C10	0.056	0.007	0.813	0.125	0.000
C	C11	0.287	0.011	0.000	0.702	0.000
C	C12	0.264	0.000	0.726	0.000	0.009
C	C13	0.350	0.190	0.440	0.000	0.020
C	C14	0.450	0.055	0.275	0.000	0.220
C	C15	0.616	0.059	0.293	0.032	0.000

*Set B simulated communities highlighted in blue are shown in Figure 1.
