# Supplementary material for: MG-MLST: Characterizing the Microbiome at the Strain Level in Metagenomic Data
Source: Microorganisms. 2020 May 8;8(5):684. doi: 10.3390/microorganisms8050684 (PMC7284976; doi:10.3390/microorganisms8050684)
Supplement: Supplementary file 1 [file microorganisms-08-00684-s001.zip › MLSTPaper-SupplementaryTableS3_final.pdf]

Table S3. Pearson's correlations of the *P. acnes* strain composition in each sample between different methods.

Healthy	H01	H02	H03	H04	H05	H06	H07	H08	H09	H10	H11	H12	H13
**Comparison to 16S Clone Library**													
**16S**	1.000	1.000	1.000	1.000	1.000	1.000	1.000	1.000	1.000	1.000	1.000	1.000	1.000
**Aarhus**	0.983	0.985	0.994	0.993	0.992	0.791	0.666	0.483	0.292	-0.069	-0.278	-0.312	-0.510
**Belfast**	0.982	0.998	0.999	0.997	0.996	0.667	0.926	0.734	0.666	-0.077	-0.281	-0.294	0.592
**Aarhus-Belfast**	0.992	0.988	0.997	0.997	0.998	0.608	0.950	0.974	0.989	0.999	0.999	0.999	0.410
**454**	0.996	-	-	-	-	-	0.573	-	-	-	-	-	-
**Comparison to MG-MLST (Aarhus)**													
**16S**	0.983	0.985	0.994	0.993	0.992	0.791	0.666	0.483	0.292	-0.069	-0.278	-0.312	-0.510
**Aarhus**	1.000	1.000	1.000	1.000	1.000	1.000	1.000	1.000	1.000	1.000	1.000	1.000	1.000
**Belfast**	0.997	0.975	0.996	0.995	0.989	0.937	0.813	0.530	0.146	0.008	-0.234	-0.263	0.246
**Aarhus-Belfast**	0.998	0.961	1.000	0.998	0.997	0.931	0.775	0.543	0.410	-0.029	-0.242	-0.276	0.406
**454**	0.995	-	-	-	-	-	0.986	-	-	-	-	-	-
**Comparison to MG-MLST (Belfast)**													
**16S**	0.982	0.998	0.999	0.997	0.996	0.667	0.926	0.734	0.666	-0.077	-0.281	-0.294	0.592
**Aarhus**	0.997	0.975	0.996	0.995	0.989	0.937	0.813	0.530	0.146	0.008	-0.234	-0.263	0.246
**Belfast**	1.000	1.000	1.000	1.000	1.000	1.000	1.000	1.000	1.000	1.000	1.000	1.000	1.000
**Aarhus-Belfast**	0.998	0.986	0.998	0.999	0.993	0.995	0.995	0.760	0.678	-0.075	-0.305	-0.322	0.978
**454**	0.991	-	-	-	-	-	0.758	-	-	-	-	-	-
**Comparison to MG-MLST (Aarhus-Belfast)**													
**16S**	0.992	0.988	0.997	0.997	0.998	0.608	0.950	0.974	0.989	0.999	0.999	0.999	0.410
**Aarhus**	0.998	0.961	1.000	0.998	0.997	0.931	0.775	0.543	0.410	-0.029	-0.242	-0.276	0.406
**Belfast**	0.998	0.986	0.998	0.999	0.993	0.995	0.995	0.760	0.678	-0.075	-0.305	-0.322	0.978
**Aarhus-Belfast**	1.000	1.000	1.000	1.000	1.000	1.000	1.000	1.000	1.000	1.000	1.000	1.000	1.000
**454**	0.998	-	-	-	-	-	0.717	-	-	-	-	-	-

Acne	A01	A02	A03	A04	A05	A06	A07	A08	A09	A10	A11	A12	A13
**Comparison to 16S Clone Library**													
**16S**	1.000	1.000	1.000	1.000	1.000	1.000	1.000	1.000	1.000	1.000	1.000	1.000	1.000
**Aarhus**	0.999	0.999	0.997	0.999	0.986	0.979	0.964	0.961	0.937	0.745	0.740	0.055	-0.185
**Belfast**	0.998	0.998	0.993	0.994	0.984	0.990	0.999	0.958	0.901	0.962	0.592	0.265	-0.253
**Aarhus-Belfast**	1.000	0.999	0.997	1.000	0.998	0.998	0.991	0.962	0.920	0.962	0.713	0.240	0.706
**454**	1.000	-	0.999	-	-	-	-	-	-	0.895	-	-	-0.106
**Comparison to MG-MLST (Aarhus)**													
**16S**	0.999	0.999	0.997	0.999	0.986	0.979	0.964	0.961	0.937	0.745	0.740	0.055	-0.185
**Aarhus**	1.000	1.000	1.000	1.000	1.000	1.000	1.000	1.000	1.000	1.000	1.000	1.000	1.000
**Belfast**	0.994	1.000	0.988	0.989	0.981	0.975	0.973	0.975	0.780	0.555	0.920	0.937	-0.293
**Aarhus-Belfast**	0.998	1.000	0.995	0.999	0.974	0.990	0.991	0.992	0.801	0.657	0.975	0.970	0.501
**454**	0.999	-	0.999	-	-	-	-	-	-	0.897	-	-	0.826
**Comparison to MG-MLST (Belfast)**													
**16S**	0.998	0.998	0.993	0.994	0.984	0.990	0.999	0.958	0.901	0.962	0.592	0.265	-0.253
**Aarhus**	0.994	1.000	0.988	0.989	0.981	0.975	0.973	0.975	0.780	0.555	0.920	0.937	-0.293
**Belfast**	1.000	1.000	1.000	1.000	1.000	1.000	1.000	1.000	1.000	1.000	1.000	1.000	1.000
**Aarhus-Belfast**	0.999	1.000	0.998	0.993	0.979	0.991	0.995	0.995	0.999	0.943	0.981	0.991	-0.163
**454**	0.998	-	0.994	-	-	-	-	-	-	0.802	-	-	-0.139
**Comparison to MG-MLST (Aarhus-Belfast)**													
**16S**	1.000	0.999	0.997	1.000	0.998	0.998	0.991	0.962	0.920	0.962	0.713	0.240	0.706
**Aarhus**	0.998	1.000	0.995	0.999	0.974	0.990	0.991	0.992	0.801	0.657	0.975	0.970	0.501
**Belfast**	0.999	1.000	0.998	0.993	0.979	0.991	0.995	0.995	0.999	0.943	0.981	0.991	-0.163
**Aarhus-Belfast**	1.000	1.000	1.000	1.000	1.000	1.000	1.000	1.000	1.000	1.000	1.000	1.000	1.000
**454**	1.000	-	0.997	-	-	-	-	-	-	0.877	-	-	0.410
