# Supplementary material for: MG-MLST: Characterizing the Microbiome at the Strain Level in Metagenomic Data
Source: Microorganisms. 2020 May 8;8(5):684. doi: 10.3390/microorganisms8050684 (PMC7284976; doi:10.3390/microorganisms8050684)
Supplement: Supplementary file 1 [file microorganisms-08-00684-s001.zip › MLSTPaper-SupplementaryTableS4_final.pdf]

Table S4. Marker SNPs for each allele of the MLST genes.

Allele	Marker SNPs for the allele
fba 2	RT1 (Default)
fba 3	C2137666A, A2137717G
fba 4	A2137717G
fba 5	A2137717G, C2137780T
fba 7	A2137702G, A2137717G, G2137837A, G2137896A
fba 8	A2137702G, A2137717G, G2137837A, G2137871A, G2137896A
fba 9	A2137702G, A2137717G, G2137759A, G2137837A, G2137896A
lac 4	RT1 (Default)
lac 5	T16011C
lac 7	A15694G, C15765T, C15772A, T15904G, C15939T, A15964G, T16011C, G16042A
lac 9	G16041T
lac 10	A15694G, C15765T, C15772A, C15846G, T15904G, C15939T, A15964G, T16011C, G16042A
recA 2	C1280249T
recA 5	RT1 (Default)
recA 6	A1280354G
recA 10*	C1280809T
zno 5	C1207754T
zno 6	RT1 (Default)
zno 7	G1207638T, C1207642T
zno 9	C1207624G, C1207642T
zno 11	C1207642T
zno 14	G1207344A, C1207348T, A1207350G, G1207368A, A1207374T, C1207376T, C1207377T, G1207434C, A1207440G, T1207447C, C1207470G, A1207534G, A1207543G, T1207576C, T1207619C, C1207642T, T1207645G, C1207647T, A1207677G, A1207692G, C1207707T
zno 15	C1207642T, G1207703C
zno 16	C1207642T, T1207683G
zno 17*	C1207348T, A1207374C, T1207404C, A1207440G, T1207447C, A1207479G, T1207506C, A1207534G, C1207538T, G1207563A, C1207572A, C1207573T, C1207580T, A1207605G, T1207619C, C1207623T, G1207635A, C1207642T, T1207645G, A1207677G, T1207686C, A1207692G, G1207693A
aroE 1	RT1 (Default)
aroE 5	G1087614A
aroE 8*	G1087713A
aroE 9*	G1087374A, T1087421C, C1087589T, G1087595A, G1087614A, G1087703A
aroE 15	T1087319C, C1087329T, T1087349C, T1087357C, G1087391A, A1087394G, A1087397G, C1087398T, A1087415G, T1087421C, T1087432C, C1087448T, G1087467A, C1087477T, G1087490C, C1087529T, C1087546T, A1087550C, G1087565A, C1087620T
aroE 16*	C1087687T
aroE 17	T1087319C, C1087329T, T1087349C, T1087357C, G1087391A, A1087394G, A1087397G, C1087398T, A1087415G, T1087421C, T1087432C, C1087448T, G1087467A, C1087477T, G1087490C, C1087529T, C1087546T, A1087550C, G1087565A
camp2 1	RT1 (Default)
camp2 2	A1603253C, T1603319A, G1603349A, C1603414T
camp2 4	G1602992T, A1603253C, T1603319A, G1603349A, C1603414T
camp2 5*	A1603253C, T1603319A, G1603349A, C1603414T, T1603441C
camp2 6	A1603253C
camp2 7	T1603047C, A1603253C, T1603319A, G1603349A, C1603414T
camp2 8	T1603047C, A1603253C, T1603319A, G1603349A, C1603414T, G1603472C
camp2 9	A1603253C, T1603319A, G1603349A, C1603414T, C1603604T

camp2 10	C1602880T, A1602904G, G1602990A, C1602994A, C1603006T, C1603009G, A1603026G, G1603119A, T1603138C, C1603141T, T1603168G, A1603208G, C1603231G, A1603253C, G1603384A, T1603441C, G1603447A, A1603466G, G1603528C, A1603556G, A1603573C, T1603579C, C1603627T, C1603630T, C1603636T
camp2 12	C1602880T, A1602904G, G1602990A, C1602994A, C1603006T, C1603009G, A1603026G, T1603138C, C1603141T, T1603168G, A1603208G, C1603231G, A1603253C, G1603384A, T1603441C, G1603447A, A1603466G, G1603528C, A1603556G, A1603573C, T1603579C, C1603627T, C1603630T, C1603636T
camp2 13	C1602880T, A1602904G, G1602990A, C1602994A, C1603006T, C1603009G, A1603026G, T1603138C, C1603141T, G1603163A, T1603168G, A1603208G, C1603231G, A1603253C, G1603384A, T1603441C, G1603447A, A1603466G, G1603528C, A1603556G, A1603573C, T1603579C, C1603627T, C1603630T, C1603636T
camp2 14*	A1603253C, A1603315C
guaA 1	T1879647C
guaA 3	RT1 (Default)
guaA 4	G1879467A, A1879485G, G1879554A, G1879569A, C1879575G, A1879599T, A1879623G, A1879638G, G1879677A, G1879701A, A1879719G, A1879722G, A1879728G, G1879740A, G1879761A, A1879779G, A1879818G, C1879926T
guaA 5	C1879553A
guaA 8*	G1879545A, A1879595G, T1879636C, G1879902A
guaA 13	G1879467A, C1879553A
guaA 15*	G1879558A
guaA 16	G1879467A, A1879485G, G1879551A, G1879554A, G1879569A, C1879575G, A1879599T, A1879623G, A1879638G, G1879677A, G1879701A, A1879719G, A1879722G, A1879728G, G1879740A, G1879761A, A1879779G, A1879818G, C1879926T
tly 1	RT1 (Default)
tly 2	C885651T, T886334G
tly 3	T885868C
tly 4	C885877T
tly 5	C885651T, T885884G, G885901T, T886334G
tly 6	C885886T, A885887T, A885891C, T885894C, T885897G
tly 8	T885943C, T886334G
tly 9	A885891C, T885943C, T886334G
tly 10	C885602T, G885606A, T885646C, T885673C, A885745G, T885751C, G885755A, C885769T, G885798C, A885799T, G885856A, C885880T, T885943C, G885959A, A886024G, G886046A, G886114T, C886148T, G886216T, T886334G, C886350A
tly 14*	A885745G, T885943C, T886334G

Alleles marked with an asterisk (*) are only considered and included when using the combined Aarhus-Belfast scheme, where strains with these alleles are included in the "Learning Sample" set.
